# Prognostic value of 12-month response to therapy in pediatric patients with differentiated thyroid cancer

**DOI:** 10.1007/s12020-023-03309-7

**Published:** 2023-01-24

**Authors:** Emilia Zampella, Leandra Piscopo, Mariarosaria Manganelli, Fabio Volpe, Carmela Nappi, Valeria Gaudieri, Leonardo Pace, Martin Schlumberger, Alberto Cuocolo, Michele Klain

**Affiliations:** 1 Department of Advanced Biomedical Sciences, University Federico II, Naples, Italy; 2 Department of Medicine, Surgery and Dentistry, University of Salerno, Salerno, Italy; 3 Consultant, Department of Advanced Biomedical Sciences, University Federico II, Naples, Italy

**Keywords:** Differentiated thyroid carcinoma, ^131^I therapy, Prognosis, Pediatric patients

## Abstract

**Purpose:**

In pediatric patients with differentiated thyroid cancer (DTC) we assessed the prognostic value of the 12-month response to therapy after initial treatment with surgery and radioactive iodine (RAI).

**Methods:**

We retrospectively evaluated 94 pediatric patients with DTC, treated with surgery and RAI who were initially classified as low, intermediate or high risk of relapse of disease according to the American Thyroid Association (ATA) guidelines. Twelve months after RAI administration the response to therapy was assessed by serum thyroglobulin (Tg) measurement and neck ultrasound and patients were classified as having excellent response (ER) or no-ER.

**Results:**

At the 12 months evaluation, 62 (66%) patients had ER and 32 (34%) no-ER. During a mean follow-up time of 86 months (range 9–517), 19 events occurred (20% cumulative event rate). Events occurred more frequently in younger patients (*p* < 0.05), in those at ATA intermediate/high risk (*p* < 0.01) and with a pre-RAI therapy Tg level > 10 ng/mL (*p* < 0.001), and in those with no-ER (*p* < 0.001). At multivariate analysis, the evidence of no-ER was the only independent predictor of events.

**Conclusion:**

In pediatric patients with DTC, the response to therapy evaluated 12 months after initial treatment has an independent prognostic impact and is able to predict mid-term outcome. Patients with no-ER at 12 months after RAI therapy should be closely followed-up.

## Introduction

Differentiated thyroid cancer (DTC) is rare in children [1, 2]. The initial treatment consists in surgery followed by radioactive iodine (RAI) therapy in selected patients [3]. DTC usually presents as a more extended disease in children and adolescents than in adult patients, with extensive regional nodal involvement and more frequent lung metastases, but the survival rate at 30 years is close to 90% [4–6]. The rate of recurrence is high in pediatric patients, in particular in those with high post-operative serum thyroglobulin (Tg) values [7, 8].

The American Thyroid Association (ATA) risk stratification classification may help to recognize at the time of initial treatment patients with a high risk of persistent or recurrent disease [9]. On the other hand, the extent of nodal disease is defined according to central or lateral location of lymph node involvement [9]. To refine this classification some authors considered the criteria used in the adult ATA guidelines, such as the number and size of the metastatic lymph nodes [7, 10, 11]. This modified ATA risk stratification was able to predict the outcome in pediatric DTC patients [10, 11]. In addition, a dynamic risk stratification system based on biochemical and imaging data that evaluates the response to initial therapy at 12 months predicted the outcome in adult patients [12, 13]. However, this prognostic approach has not been fully addressed in children.

The aim of this study was to evaluate the prognostic role of the response to therapy assessed at 12 months after initial treatment in pediatric patients with DTC.

## Materials and methods

### Study population

We retrospectively evaluated 122 pediatric DTC patients (age < 18 years) referred to our Department between 1992 and 2010. All patients underwent a total thyroidectomy, with or without central and/or lateral neck dissection, followed by RAI therapy. Before RAI administration, L-thyroxine was discontinued until the serum thyroid stimulating hormone (TSH) level has increased above 30 mIU/l. At the time of RAI, serum thyroglobulin (Tg) was determined and a ^131^I activity according to the body weight and the amount of disease (37 to 111 MBq/Kg) was administered. From 1992 to 1996, serum Tg levels were determined by an immunoradiometric assay (Dynotest Tg, Henning, Berlin, Germany) with a sensitivity of 1 ng/mL. From 1997, Tg was determined by a chemiluminescence system (Immulite, Diagnostic Products Corp, Los Angeles, CA, USA) with a detection limit of 0.2 ng/mL. According to post-operative Tg values obtained at the time of RAI therapy, patients were categorized into two groups: > 10 ng/ml and ≤ 10 ng/ml [7, 14]. Five to seven days after ^131^I administration, a post-therapy whole body scan (WBS) was performed as previously described using a dual-head gamma camera (E.CAM, Siemens Medical Systems, Hoffman Estates, IL, USA) equipped with thick crystals and high energy collimators [15].

### ATA initial risk stratification

The risk of persistence or recurrence was initially estimated according to histopathological findings and the results of ^131^I post-therapeutic WBS, based on ATA pediatric guidelines [9]. Patients were classified as low risk (disease grossly confined to the thyroid, with N0 or Nx disease or with incidental N1a metastasis), intermediate risk (extensive N1a or minimal N1b disease) or high risk (extensive N1b disease or T4 tumor with gross tumor extension beyond the thyroid capsule, with or without distant metastasis). To consider nodal involvement as extensive, we used the number and size of lymph nodes, for N1b > 5 or any lymph node metastases ≥ 3 cm or the presence of any clinically detected lymph node metastases, according to the ATA adult guidelines [10, 11].

### Therapy response evaluation

The response to therapy at 12 months after RAI administration was assessed with serum Tg measurement obtained on LT4 treatment or following thyroid hormone withdrawal, neck ultrasound and imaging findings. According to the 2015 ATA guidelines for adults [10], definitions of response to therapy were: (1) excellent response (ER), negative imaging and either Tg < 0.2 ng/mL on LT4 treatment or TSH-stimulated Tg < 1 ng/mL; (2) indeterminate response, non-specific findings on imaging studies and Tg levels on LT4 treatment that are detectable but < 1 ng/mL or stimulated Tg levels between 1 and 10 ng/mL or stable or declining titer of anti-Tg antibodies over time; (3) biochemical incomplete response, negative imaging and Tg ≥ 1 ng/mL on LT4 treatment or stimulated Tg ≥ 10 ng/mL or rising titer of anti-Tg antibody over time (4) structural incomplete response, structural evidence of disease with any level of serum Tg or of anti-Tg antibodies. Patients without neck ultrasound at the 12-month evaluation were excluded from the study.

### Follow-up

After the evaluation at 12-months, all patients were followed every 6–12 months with serum Tg determinations (on L-thyroxine and in some patients off L-thyroxine therapy) and with imaging procedures. Disease status was recorded at each evaluation. Disease-free survival was measured from the date of surgery to the first observation of structural persistent disease, structural recurrent disease or additional treatment (e.g., surgery, RAI). Structural disease was defined as “persistent” in patients that one year after treatment had detectable Tg, detectable Tg antibodies or abnormal neck ultrasound, and as “recurrent” in patients who achieved remission but then developed detectable Tg, detectable Tg antibodies, or abnormal neck ultrasound. Suspicious nodal abnormalities at neck ultrasonography were confirmed by fine needle aspiration cytology and Tg determination in the aspirate fluid, histology or presence of RAI uptake; uptake in the thyroid bed at post-therapy WBS was considered a structural disease only when it corresponded to abnormal findings at neck ultrasonography [10] Patients last known to be alive and without structural disease were censored at the date of last contact.

### Statistical analysis

Continuous data are expressed as mean ± standard deviation and categorical data as percentage. Student’s two-sample *t* test and χ^2^ test were used to compare the differences in continuous and categorical variables, respectively. Univariate and multivariate logistic regression analyses were performed to identify the variables associated with 12 months response to initial treatment. Hazard ratios with 95% confidence intervals (CI) were also calculated by univariate and multivariate Cox regression analyses. Variables showing a *p*-value < 0.05 at univariate analysis were considered for multivariate analysis. Disease free survival analysis was performed using the Kaplan-Meier method and log-rank test. Statistical analysis was performed with Stata 12 software (StataCorp, College Station, Texas USA).

## Results

Among 122 pediatric patients with DTC, 28 without serum Tg determinations and/or neck ultrasound at the 12-months evaluation were excluded, leaving 94 subjects for the analysis. At initial evaluation, 62 (66%) patients were classified as ATA low risk, 17 (18%) as ATA intermediate risk and 15 (16%) as ATA high risk. Of these latter patients at high risk, 3 had lung metastases at post-therapy WBS.

At the 12 months evaluation, 62 (66%) patients had ER to initial therapy and 32 (34%) no-ER (Table 1). Among the no-ER patients, 11 were classified as indeterminate response, 17 as biochemical incomplete response and 4 as structural incomplete response for lung metastases (*n* = 3) or for lymph node metastases (N1b) confirmed by fine needle aspiration biopsy (*n* = 1). The 3 patients with lung metastases underwent a second RAI treatment and the patient with lymph node metastases was treated with surgery. Among 32 no-ER patients, 15 (47%) were initially classified as ATA low risk and 17 (53%) as ATA intermediate/high risk. Serum Tg at the time of RAI treatment was > 10 ng/mL in 25 (78%) no ER patients. Twelve (37%) patients were younger than 14 years at the time of initial treatment (Table 1). Among 62 patients initially classified as low ATA risk, pre-therapy Tg values and prevalence of serum Tg > 10 ng/mL were significantly higher in patients with no-ER (*n* = 15) as compared to those with ER (*n* = 47) (both *p* < 0.01) while the other baseline findings were comparable among the two groups (Supplementary Table). At multivariate logistic regression analysis, a pre-therapy Tg > 10 ng/mL was the only independent predictor of no-ER (*p* < 0.001) (Table 2).

**Table 1:** Baseline characteristics according to the 12-months response to initial treatment

	All patients (*n* = 94)	No-ER (*n* = 32)	ER (*n* = 62)	*p*-value
Age (years)	16 ± 2	15 ± 3	16 ± 2	0.34
Age ≤ 14years, *n* (%)	23 (25)	12 (37)	11 (1)	< 0.05
Male gender, *n* (%)	24 (26)	12 (37)	12 (20)	0.06
ATA risk categories				
Low risk, *n* (%)	62 (66)	15 (47)	47 (76)	< 0.01
Intermediate/high risk, *n* (%)	32 (34)	17 (53)	15 (24)	< 0.01
Follicular type, *n* (%)	6 (6)	4 (12)	2 (3)	0.08
Tumor size > 2 cm, *n* (%)	58 (62)	25 (78)	33 (53)	< 0.05
Neck dissection, *n* (%)	66 (70)	25 (78)	41 (66)	0.23
Lymph node involvement, *n* (%)	57 (61)	24 (75)	33 (53)	< 0.05
Time interval surgery/RAI therapy (days)	135 ± 159	142 ± 153	132 ± 162	0.77
Administered ^131^I activity (MBq)	3182 ± 958	3182 ± 999	3182 ± 962	0.96
Pre-therapy Tg (ng/ml)	31 ± 61	63 ± 81	13 ± 29	< 0.001
Pre-therapy Tg > 10 ng/ml, *n* (%)	39 (41)	25 (78)	14 (23)	< 0.001
Uptake at WBS, *n* (%)	92 (98)	31 (97)	61 (98)	0.63
Neck, *n*		31	61	
Extra-neck, *n*		3	0	

Data are presented as mean ± SD or number and percentage (%)

*Tg* thyroglobulin, *WBS* post-therapy whole body scan

**Table 2:** Univariate and multivariate logistic regression analyses with 12 months no-ER as dependent variable

	Univariate		Multivariate	
	Odds ratio (95% CI)	*p-*value	Odds ratio (95% CI)	*p-*value
Age ≤ 14 years	2.42 (0.91–6.45)	0.07		
Intermediate/high risk	3.55 (1.43–8.78)	< 0.001	2.50 (0.87–7.15)	0.09
Tg > 10 ng/mL	12.2 (4.38–34.2)	< 0.001	7.04 (3.74–30.3)	< 0.001

*Tg* Thyroglobulin obtained following thyroid hormone withdrawal before the RAI administration

### Predictors of outcome

During a median follow-up of 86 months (range 9–517 months), 19 patients experienced an event occurred (20% cumulative event rate). Twelve of the patients with event required additional RAI therapy. In particular, 2 patients were treated for recurrence in the thyroid bed, 4 for persistent disease in the thyroid bed, 4 for persistent disease in both thyroid bed and lymph nodes, and 2 for lung metastases. The remaining 7 patients with event underwent both additional surgery and RAI therapy for nodal disease (6 patients for persistent disease and 1 for recurrent disease).

Patients with events were younger at initial treatment (*p* < 0.05), had a higher prevalence of ATA intermediate/high risk (*p* < 0.01) and pre-therapy Tg > 10 ng/mL (*p* < 0.001) (Table 3). The rate of events was significantly higher in the 32 patients with no-ER (*n* = 16, 50%) at 12 months as compared to the 62 patients with ER (*n* = 3; 5%) (*p* < 0.001). Individual characteristics of the 3 patients with ER who had recurrent disease are reported in Table 4. At multivariate Cox analysis (Table 5), no-ER remained the only independent predictor of events (*p* < 0.001).

**Table 3:** Baseline characteristics according to the occurrence of events

	Event (*n* = 19)	No event (*n* = 75)	*p*-value
Age (years)	15 ± 3	16 ± 2	0.62
Age ≤ 14years, *n* (%)	8 (40)	15 (20)	< 0.05
Male gender, *n* (%)	6 (32)	18 (24)	0.49
*ATA risk categories*			
Low risk, *n* (%)	6 (32)	56 (75)	< 0.01
Intermediate/high risk, *n* (%)	13 (68)	19 (25)	< 0.01
Follicular type, *n* (%)	2 (11)	4 (5)	0.41
Tumor size > 2 cm, *n* (%)	14 (74)	44 (59)	0.23
Neck dissection, *n* (%)	16 (84)	50 (67)	0.14
Lymph node involvement, *n* (%)	16 (84)	41 (55)	< 0.05
Time interval surgery/RAI therapy (days)	125 ± 145	139 ± 162	0.74
Administered ^131^I activity (MBq)	3367 ± 814	3136 ± 984	0.36
Pre-therapy Tg (ng/ml)	74 ± 90	19 ± 45	< 0.001
Pre-therapy Tg > 10 ng/ml, *n* (%)	16 (84)	23 (31)	< 0.001
Uptake at WBS, *n* (%)	18 (95)	74 (99)	0.29
Neck, *n*	18	74	
Extra-neck, *n*	3	0	

Data are presented as mean ± SD or number and percentage (%)

*Tg* Thyroglobulin, *WBS* Post-therapy whole body scan

**Table 4:** Individual characteristics of the ER patients with events during follow-up

	Patient 1	Patient 2	Patient 3
RAI therapy			
ATA risk categories	Low	Intermediate	Low
Tg (ng/ml)	19	89	0.7
Uptake at WBS	Neck	Neck	−
12 months evaluation			
Tg (ng/ml)	0.5	0.6	0.1
Ultrasound	−	−	−
Recurrent disease during follow-up			
Site of recurrence	Thyroid bed	Thyroid bed	Lymph nodes
Time from 12 months evaluation (months)	29	52	139
Tg (ng/ml)	23	10	2
Treatment	RAI	RAI	Surgery + RAI

*Tg* Thyroglobulin, *WBS* Post-therapy whole body scan, *ER* Excellent response

**Table 5:** Univariate and multivariate predictors of events

	Univariate		Multivariate	
	Hazard ratio (95% CI)	*p*-value	Hazard ratio (95% CI)	*p*-value
Age ≤ 14 years	2.38 (0.95–5.92)	0.06		
Intermediate -High ATA risk	4.62 (1.75–12.1)	< 0.01	2.22 (0.79–6.22)	0.13
Tg > 10 ng/mL	9.25 (2.69–31.8)	< 0.001	2.35 (0.51–30.3)	0.27
No-ER vs. ER	14.2 (4.11–48.7)	< 0.001	6.99 (1.61–30.3)	< 0.001

*Tg* thyroglobulin obtained following thyroid hormone withdrawal before the RAI administration, *ER* excellent response

**Fig. 1:**
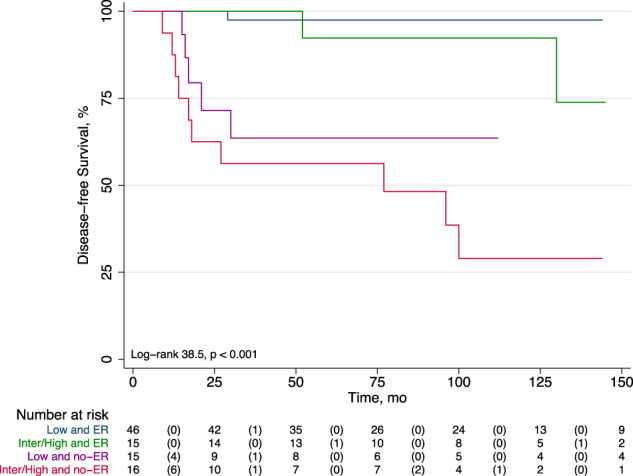
Disease free survival by Kaplan-Meier according to 12-month response to therapy and ATA risk. Patients with no-ER and intermediate/high ATA risk had the worst outcome compared to the other groups (*p* < 0.001)

**Fig. 2:**
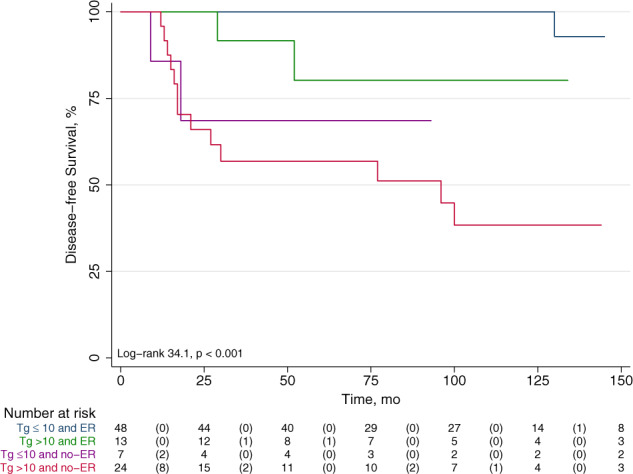
Disease free survival by Kaplan-Meier according to 12-month response to therapy and post-operative serum Tg levels. Patients with no-ER and pre-therapy Tg > 10 ng/mL had the worst outcome compared to the other groups (*p* < 0.001)

At Kaplan Meier analysis, the disease-free survival was lower in patients with no-ER as compared to those with ER (177 ± 32 vs. 477 ± 23 months, *p* < 0.001). The worst prognosis was observed in patients with no-ER and intermediate/high ATA risk (Fig. 1) and in patients with no-ER and pre-therapy Tg > 10 ng/mL (Fig. 2) (both *p* < 0.001).

## Discussion

In this retrospective analysis, we found that the response to initial treatment evaluated at 12 months with serum Tg determination and neck ultrasound, is able to predict the mid-term outcome of pediatric DTC patients treated with surgery and RAI.

At initial diagnosis, pediatric patients with DTC present with more extensive disease as compared to adults [1–4]. Moreover, in children lung metastases are more frequently observed and persist also after multiple RAI therapies [16, 17]. However, death is uncommon and usually occurs several decades after diagnosis [18]. In children with DTC the survival rate at 30 years close to 90% [1–4, 19]. However, the rate of recurrence is higher in pediatric than in adult patients, in particular in those with multifocal papillary thyroid cancer, with gross tumor extension beyond the thyroid capsule and in those with extensive lymph node involvement [20]. Pediatric patients with pre-RAI therapy Tg levels > 10 ng/ml have a higher risk of structural persistent disease, in particular those classified as ATA intermediate risk [8, 9, 21]. The initial risk stratification in pediatric patients with DTC is performed according to pediatric ATA guidelines, where the extent of nodal disease is defined according to central or lateral location of lymph node involvement [9]. We used a refined classification considering the criteria used in the adult ATA guidelines, such as the number and size of the metastatic lymph nodes [7, 11], and this modified ATA risk stratification was able to predict outcome in a cohort of 260 children with DTC [11]. In adult patients the dynamic risk stratification has been proposed to evaluate the response to therapy during follow-up. The addition of laboratory and imaging findings obtained during the first 12–24 months after treatment, can improve the initial risk assessment in adult [13] and also in pediatric patients [8, 21].

In this retrospective analysis of 94 pediatric patients, 62 (66%) were initially classified as ATA low risk. At 12 months evaluation, most (76%) of these low risk patients, had an ER, in agreement with the series of Sapuppo et al. [11]. However, the other 15 (24%) low-risk patients had no-ER, suggesting that a further stratification in these patients is needed.

An incomplete primary surgery may explain these findings, also considering that patients with no-ER showed higher pre-therapy Tg values as compared to those who had ER. Incomplete primary surgery could be due to non-accurate preoperative staging, including ineffective evaluation of lymph node status and local extension. The identification of persistent disease after surgery may be challenging, considering that local postoperative inflammation can affect neck ultrasound findings and RAI uptake in the thyroid bed is frequently observed at post-therapy WBS scan. In our population 92 of 94 patients showed neck uptake; moreover, it should be considered that at the time of treatment single photon emission computed tomography/computed tomography/CT) hybrid technology was not available.

From our results it emerged that, despite no-ER was more frequent in younger patients, the presence of pre-therapy Tg values > 10 ng/mL was the only independent predictor of no-ER. Predicting 12 months response to treatment is crucial, considering that the presence of no-ER is associated with unfavorable outcome. In our series, the rate of events was higher in no-ER patients, and the presence of no-ER remained the only independent predictor of outcome at multivariable analysis. Interestingly, lung metastases were observed in only 3 patients at diagnosis. Despite the evidence of structural incomplete response at 12-month evaluation and the need for a second RAI treatment, at the end of follow-up two of these patients had an excellent response to treatment. The complete remission in patients with lung lesions is uncommon, despite it has been suggested that an early diagnosis and the absence of defined nodules may influence the response to therapy [22]. In our series, the 2 patients whit lung metastases who achieved an excellent response had a micronodular pattern, and the absence of nodules may have resulted in a more effective response to RAI. In our population the rate of persistent disease was higher in patients with biochemical incomplete response at 12 months. Moreover, 11 patients showed also indeterminate response and 9 of them ended the follow-up without any evidence of disease.

In adult patients with low-risk thyroid cancer, the probability of persistent disease is low in patients with low or undetectable post-operative TSH stimulated Tg level, and the benefits of RAI administration have been recently questioned [23, 24]. This approach should be further investigated in in low risk children in order to reduce their irradiation whenever it is not beneficial. On the contrary, in patients initially at higher risk, the evaluation at 12-months might better identify patients in whom a more aggressive follow-up is needed.

## Conclusions

Our data suggest that in pediatric patients with DTC the response to therapy evaluated 12 months after initial treatment has an independent prognostic impact and is able to predict mid-term outcome. Patients with no-ER at 12 months after RAI therapy should be closely followed-up.

## Supplementary Information

Supplementary Information
