# Supplementary material for: Prognostic value of 12-month response to therapy in pediatric patients with differentiated thyroid cancer
Source: Endocrine. 2023 Jan 24;80(3):612–8. doi: 10.1007/s12020-023-03309-7 (PMC10199830; doi:10.1007/s12020-023-03309-7)
Supplement: Supplementary file 1 — Supplementary Information [file 12020_2023_3309_MOESM1_ESM.docx]

**Supplementary Table**. Baseline characteristics of 62 patients initially classified as low ATA risk according to the 12-months response to initial treatment

|  | All patients  (*n* = 62) | No-ER  (*n* = 15) | ER  (*n* = 47) | *p* value |
| --- | --- | --- | --- | --- |
| Age (years) | 15 ± 2 | 15 ± 3 | 16 ± 2 | 0.40 |
| Age ≤14years, *n* (%) | 12 (19) | 5 (33) | 7 (15) | 0.12 |
| Male gender, *n* (%) | 15 (24) | 5 (33) | 10 (21) | 0.34 |
| Follicular type, *n* (%) | 4 (6) | 2 (13) | 2 (5) | 0.21 |
| Tumor size >2 cm, *n* (%) | 33 (53) | 10 (67) | 23 (49) | 0.23 |
| Neck dissection, *n* (%) | 34 (55) | 8 (53) | 26 (55) | 0.89 |
| Lymph node involvement, *n* (%) | 26 (42) | 8 (53) | 18 (38) | 0.30 |
| Time interval surgery/RAI therapy (days) | 141 ± 83 | 110 ±82 | 151 ± 181 | 0.39 |
| Administered ^131^I activity (MBq) | 3071 ± 962 | 2997 ± 1073 | 3108 ± 999 | 0.72 |
| Pre-therapy Tg (ng/ml) | 15 ± 27 | 36 ± 47 | 8 ± 12 | <0.01 |
| Pre-therapy Tg >10 ng/ml, *n* (%) | 20 (32) | 10 (67) | 10 (21) | <0.01 |
| Uptake at WBS, *n* (%) | 61 (98) | 15 (100) | 46 (98) | 0.57 |
| Neck, *n* |  | 15 | 46 |  |
| Extra-neck, *n* |  | 0 | 0 |  |

Data are presented as mean ± SD or number and percentage (%)

*Tg* thyroglobulin, *WBS* post-therapy whole body scan
